# Air temperature and humidity impact out-of-hospital-cardiac-arrests in Germany: A 10-year cohort study from the German Resuscitation Registry

**DOI:** 10.1016/j.resplu.2024.100750

**Published:** 2024-08-24

**Authors:** Maximilian Burger, Patrick Ristau, Andreas Bohn, Matthias Fischer, Ingvild Beathe Myrhaugen Tjelmeland, Stephan Seewald, Jan-Thorsten Gräsner, Jan Wnent

**Affiliations:** a University Hospital Schleswig-Holstein, Institute for Emergency Medicine, Kiel, Germany; b University of Lübeck, Institute of Social Medicine and Epidemiology, Nursing Research Unit, Lübeck, Germany; c University Hospital Schleswig-Holstein, Dept. of Anaesthesiology and Intensive Care Medicine, Kiel, Germany; d City of Münster Fire Department, Germany; e Department of Anesthesiology, Intensive Care and Pain Medicine, University Hospital Münster, Germany; f Department of Anaesthesiology, Intensive Care Medicine, Emergency Medicine, and Pain Therapy, Alb Fils Kliniken, Göppingen, Germany; g Division of Prehospital Services, Oslo University Hospital, Oslo, Norway; h Faculty of Medicine, Institute of Clinical Medicine, University of Oslo, Oslo, Norway

**Keywords:** OHCA, Out-of-hospital-cardiac-arrest, Climate change, Public health, EMS, Resuscitation, Enviromental factors, Air temperature, Humidity, Outcome

## Abstract

**Objectives:**

This study examines the impact of temperature variations on out-of-hospital-cardiac-arrests in Germany over a decade (2010–2019). Out-of-hospital-cardiac-arrests affects 164 per 100,000 inhabitants annually in Germany, 11% survive to hospital discharge. The following study investigates days with the following characteristics: summer days, frost days, and high humidity days. Furthermore, the study explores incidence, causes, demographics, and outcomes of out-of-hospital-cardiac-arrests.

**Methods:**

Data from the German Resuscitation Registry and Meteorological Service were combined for analysis. The theory posits that temperature and humidity play a significant role in the occurrence and outcomes of out-of-hospital-cardiac-arrests, potentially triggering pre-existing health issues.

**Results:**

Findings reveal increased out-of-hospital-cardiac-arrests during frost days (6.39 up to 7.00, *p* < 0.001) monthly per 100,000 inhabitants), notably due to cardiac-related causes. Conversely, out-of-hospital-cardiac-arrests incidence decreases on summer days (6.61–5.79, *p* < 0.001 monthly per 100,000 inhabitants). High-humidity days exhibit a statistically significant increase in out-of-hospital-cardiac-arrests incidence (6.43–6.89, *p* < 0.001 monthly per 100,000 inhabitants).

**Conclusion:**

In conclusion, there’s a notable rise in out-of-hospital-cardiac-arrests incidence and worse outcomes during cold days, and a significant increase in out-of-hospital-cardiac-arrests during high-humidity days. Moreover, extreme temperature events in unaccustomed regions also elevate out-of-hospital-cardiac-arrests rates. However, the dataset lacks sufficient hot days for conclusive findings, hinting that very hot days might also affect out-of-hospital-cardiac-arrests incidence. Further research, particularly on hotter days, is essential.

No third-party funding was received for this study.

## Introduction

Sudden cardiac arrest is the third most common cause of death in Europe.^1^, ^2^, ^3^ Emergency medical services (EMS) and prehospital emergency physicians treat approximately 78 per 100,000 inhabitants with a resuscitation procedure annually, where only about 11% of the resuscitated patients subsequently leave the hospital alive in Germany.^1^ The EuReCa TWO study examined 28 participating European countries (comprising 178,879,118 inhabitants). The study revealed that cardiopulmonary resuscitation (CPR) was attempted in OHCA at a rate of 56 per 100,000 inhabitants per year in Europe (with a range of 21–91). That corresponds to a monthly incidence of 4.67 OHCA cases per 100,000 inhabitants. Furthermore, a medical cause was implicated in 91% of cases. At the scene, 64% of the individuals were found to be irreversibly deceased and were therefore not transported to the hospital. Out of the total number of patients for whom resuscitation was initiated and survival status was available, 8% survived until their hospital discharge.^2^

Although OHCA was more frequently linked to cold weather in the past,^4^ the global climate is currently changing in the warmer direction. While there are indications in the literature concerning the effect of extreme temperatures on the incidence of OHCA,^4^, ^5^, ^6^, ^7^ there is currently a lack of data regarding extreme weather events and their impact on OHCA in Germany and Europe. According to Mora et al., by 2100, approximately 75% of the world’s population will be exposed to deadly heat conditions yearly.^8^ Climate change increases the frequency and intensity of extreme weather events and changes the statistical distribution of temperature towards higher temperatures. The Intergovernmental Panel on Climate Change (IPCC) recommends the following phrase to represent the trend: “increase in the intensity and frequency of hot extremes”.^9^ As air temperature exerts a significant impact on homeostasis, both hot and cold temperature events have the potential to trigger a higher number of cardiac events through distinct pathological pathways.^10^, ^11^, ^12^, ^13^ There is evidence indicating that heat waves increase all-cause mortality,^13^, ^14^, ^15^, ^16^ the influence of heat and cold on the incidence of OHCA and the success of resuscitation has not yet sufficiently been studied in Germany. We suspect that air temperature and humidity do influence the occurrence and outcome of OHCAs.

## Material and methods

### Objectives

This study comprises a registry-based epidemiological cross-sectional cohort covering a 10-year period (01/01/2010–31/12/2019) in which data from the German Resuscitation Registry (GRR) and the German Meteorological Service (DWD) were matched.

### Inclusion and exclusion criteria

**Fig. 1:**
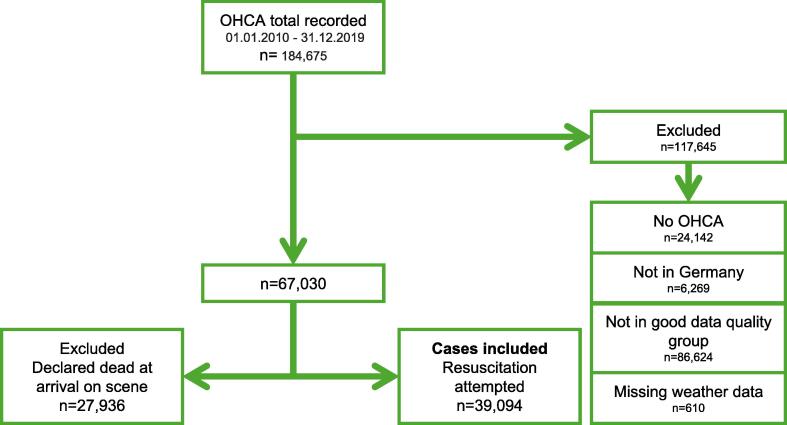
Flowchart included and excluded cases. OHCA stands for out-of-hospital cardiac arrests.

We included all OHCA (Fig. 1) in the reference EMS systems in Germany during the study period (01/01/2010–31/12/2019), where resuscitation was attempted (39,094 cases). All cases where mandatory patient data and weather data were lacking were excluded.

### Origin of data

The GRR provided the case data for this study. GRR collects data from 114 EMSs, more than 140 in-hospital medical emergency and resuscitation teams, and 90 cardiac arrest centres in Germany, Austria and Switzerland.^1^, ^17^, ^18^ The GRR primarily serves as a measurement, reporting, and quality management tool to help improve survival after cardiac arrest.

In 2022, the EMS delivering data to GRR covered a population of 32 million,^1^ and all participating EMS regions took part voluntarily. For our study, we only used data from those EMS sites that delivered high-quality data and were therefore recognised as a good data quality group in the GRR database. A good data quality group is defined according to the following criteria^1^:

Incidence of CPR of over 30 per 100,000 inhabitants per year, ROSC < 80% (to counter positive data entry bias), return of spontaneous circulation after cardiac arrest (RACA) score is calculable in >60% of cases, proportion of documented admitted patients in-hospital treatment is >30%.

The good-quality data status is calculated yearly. It covers up to 9 million inhabitants annually. Variables in GRR follow the Utstein recommendations, the standardised guidelines for uniform reporting on OHCA.^19^ The GRR data consists of two distinct datasets, namely prehospital and post-resuscitation care. The prehospital section documents logistical information, presumed aetiology, resuscitation therapy, and the initial state of the patient, whereas the data set on post-resuscitation care documents in-hospital post-resuscitation efforts, including, but not limited to, ECG, circulatory support, the cerebral performance category, and survival at both 24 h and hospital discharge. Furthermore, the GRR includes dichotomous (yes/no) questions on diagnostic or therapeutic procedures, such as percutaneous coronary intervention, targeted temperature management, ultrasound, computer tomography, and coronary artery bypass.^20^, ^21^

The German Meteorological Service (DWD) provided the weather data used in this study. The DWD is a German Federal Agency that operates the largest weather-measuring network in Germany and provides high-quality data that is both reliable and comparable. For each case, we matched the monthly and daily weather data from the nearest weather station of the respective EMS.

We used defined meteorological days as cohort dividers (Supplementary Fig. 3). A detailed description of the cases in the exposed to non-exposed group can be found in Supplementary Table 2. Summer days were defined as days when the air temperature reaches ≥25 °C, while frost days were defined when the air temperature reaches ≤0 °C. Specified meteorological days are generally known and used definitions in Germany, which are also regularly used by the German Meteorological Service. High humidity days were defined as days when the mean of the relative air humidity was >90%. This scale was used because relative air humidity above 90% by far exceeds the physiologically optimal level of humidity for humans.^22^

### Statistical analysis

Data preparation was carried out using Microsoft Excel (Microsoft Corporation, Redmond, WA, USA) and Python 3.11, while the statistical analyses were conducted with IBM SPSS, V27 (IBM, Armonk, NY, USA). Descriptive parameters such as the mean and standard deviation (SD) were calculated. We used Pearson’s chi-square, Kruskal-Wallis, and Mann-Whitney-U tests to test the null hypothesis. *P*-values <0.05 were considered significant. We also conducted a regression analysis to determine the factors influencing the correlation of monthly incidence of attempted CPR by Emergency Medical Service, where we included the most significant contributors (such as age and different monthly temperatures).

We used the cohort dividers to allocate exposed to non-exposed (Supplementary Fig. 3, Supplementary Table 2). To compare the monthly OHCA incidences of each cohort, the monthly incidence of attempted resuscitation for each case in the local area of every EMS was calculated. The mean value was used for each cohort from the monthly incidences of the cases. The resulting weighted averages were used in the monthly incidences shown.

Since various regions have different local weather phenomena, the local population could be adapted to the regional weather conditions. Thus, to measure how extreme temperature events in unaccustomed regions affects a population that is not accustomed to it, we developed an indicator (G10). Firstly, we calculated the monthly 10-year mean air temperature for every month for every EMS. Secondly, we calculated the monthly temperature difference on a case-by-case basis (e.g., April 2019–mean April), whereby 10% of the upper and lower values were used as cohort dividers (G10). Temperatures below the 10th percentile were named daily ambient air temperature-minimum (TN), temperatures above the 10th percentile were named daily ambient air temperature-maximum (TX), and the other 80% were named daily ambient air temperature (TT).

At the time of hospital admission, the conditions of admission with ROSC, ongoing CPR, or death at the scene were recorded together with the patient survival rates after 30 days. A good neurological outcome was a cerebral performance category (CPC) of 1 and 2. Sex was used to describe the patient’s biological features and was noted by the emergency physician in the field, together with the suspected cause of OHCA.

### Ethics

A study protocol was prepared and approved by the scientific advisory board of GRR (AZ 2022-02) a priori. Our study was approved by the Ethics Committee of the Medical Faculty of Kiel University (reference D 450/22). We used the STROBE Statement to report our results.^23^

## Results

During the observation period, up to 9 million inhabitants per year were covered, resulting in 39,094 cases of OHCA with resuscitation attempted to be included in this study.

The mean age of the OHCA patients was 69 (SD 95% 16.97) years. 65% of all cases were male, 35% were female, and 62% had a suspected cardiac cause of OHCA. Based on the 39,094 cases in which resuscitation was attempted, the more extensive evaluations presented in Table 1 were calculated. The overall monthly incidence was 6.50 per 100,000 inhabitants.

**Table 1:** **(a–d)** Results: ROSC stands for return-of-spontaneous-circulation. SD95% stands for the 95% confidence interval. CPR stands for cardio-pulmonary resuscitation. **a:** Summer Days. **b:** Frost Days. **c:** High Humidity Days. **d:** Days with the biggest difference from the local monthly 10-year mean air temperature were named G10. The G10 cohort ranked the temperature differences on a case-by-case basis (e.g., April 2019–mean April), 10% of the upper (TX) and lower values (TN) were used as cohort dividers.

(a) Summer Days (39,094 cases)				
	Non-extreme day (34,421 cases)	Extreme-day (4673 cases)	Missing cases	*p*-Value
Incidence per month/100,000 inhabitants	6.61 SD95%: 6.58–6.63	5.79 SD95%: 5.73–5.85	402	<0.001
Age	68.68 SD95%: 68.5–68.86	67.73 SD95%: 67.23–68.23	402	<0.001
Sex (male/female)	64.8%/35.2%	66.2%/33.8%	28	0.062
Suspected cause			0	<0.001
Cardial	61.9%	61.2%		
Trauma	2.8%	3.7%		
Hypoxia	12.5%	12.3%		
Other	9.4%	10.1%		
Unknown	13.4%	12.7%		
Hospital admission			53	0.012
Death at scene	51.6%	49.5%		
Admission with ROSC	37.5%	39.7%		
Admission with ongoing CPR	10.9%	10.8%		
Overall survival			24,241	0.935
Yes	30.0%	30.1%		
Neurological outcome			26,238	0.229
Good and moderate cerebral performance	25.00%	23.6%		
Severe disability, coma and death	75.00%	76.4%		

(b) Frost Days				
	Non-extreme day (31,330 cases)	Extreme-day (7764 cases)	Missing cases	*p*-Value
Incidence per month/100,000 inhabitants	6.39 SD95%: 6.36–6.41	7.00 SD95%: 6.95–7.05	402	<0.001
Age	68.47 SD95%: 68.28–68.66	68.97 SD95%: 68.59–69.34	402	0.037
Sex (male/female)	64.9%/35.1%	64.9%/35.1%	28	0.982
Suspected cause			0	0.012
Cardial	61.5%	62.9%		
Trauma	3.0%	2.7%		
Hypoxia	12.6%	11.8%		
Other	9.6%	9.2%		
Unknown	13.3%	13.4%		
Hospital admission			53	0.05
Death at scene	51.00%	52.5%		
Admission with ROSC	38.1%	36.6%		
Admission with ongoing CPR	10.9%	10.9%		
Overall survival			24,241	0.102
Yes	30.3%	28.7%		
Neurological outcome			26,238	0.221
Good and moderate cerebral performance	25.1%	23.9%		
Severe disability coma and death	74.9%	76.1%		

(c) High Humidity Days				
	Non-extreme day (32,315 cases)	extreme-day (6779 cases)	Missing cases	*p*-Value
Incidence per month/100,000 inhabitants	6.43 SD95%: 6.40–6.45	6.89 SD95%: 6.83–6.95	402	<0.001
Age	68.56 SD95%: 68.38–68.75	68.57 SD95%: 68.17–68.98	402	0.817
Sex (male/female)	64.9%/35.1%	65.3%/34.7%	28	0.524
Suspected cause			0	0.002
Cardial	61.4%	63.7%		
Trauma	3.0%	2.6%		
Hypoxia	12.7%	11.4%		
Other	9.5%	9.6%		
Unknown	13.4%	12.7%		
Hospital admission			53	0.022
Death at scene	51.00%	52.7%		
Admission with ROSC	38.1%	36.3%		
Admission with ongoing CPR	10.9%	10.9%		
Overall survival			24,241	0.339
Yes	29.8%	30.8%		
Neurological outcome			26,238	0.09
Good and moderate cerebral performance	24.5%	26.2%		
Severe disability, coma and death	75.5%	73.8%		

(d) G10				
	Non-extreme day (31,236 cases)	Extreme-day (TN 3943 cases/TX 3915 cases)	Missing cases	*p*-Value
Incidence per month/100,000 inhabitants	6.43 SD95%: 6.41–6.45	TN: 7.04 TX: 6.58 TN SD95%: 6.96–7.11 TX SD95%: 6.52–6.68	402	<0.001
Age	68.56 SD95%: 68.37–68.75	TN: 68.61 TX: 68.56 TN: SD95%: 68.08–69.13 TX: SD95%: 68.02–69.11	402	0.857
Sex (male/female)	65.1%/34.9%	TN: 64.0%/36.0% TX: 64.6%/35.4%	28	0.407
Suspected cause			0	0.060
Cardial	61.9%	TN: 61.7% TX: 61.3%		
Trauma	3.0%	TN: 2.9% TX: 2.6%		
Hypoxia	12.4%	TN: 12.4% TX: 13.5%		
Other	9.4%	TN: 9.5% TX: 9.9%		
Unknown	13.3%	TN: 13.5% TX: 12.7%		
Hospital admission			53	0.922
Death at scene	51.4%	TN: 51.4% TX: 50.8%		
Admission with ROSC	37.7%	TN: 37.5% TX: 38.4%		
Admission with ongoing CPR	10.9%	TN: 11.1% TX: 10.7%		
Overall survival			24,241	0.364
Yes	29.9%	TN: 29.3% TX: 31.5%		
Neurological outcome			26,238	0.032
Good and moderate cerebral performance	25.2%	TN: 24.7% TX: 21.9%		
Severe disability, coma and death	74.8%	TN: 75.3% TX: 78.1%		

The average daily temperature for the winter months (December, January, and February) was 3.1 °C and 18.2 °C for the summer months (July, August, and September), respectively. The daily average air temperature between summer and winter months differs by approximately 15 °C.

### Incidence variation

**Fig. 2:**
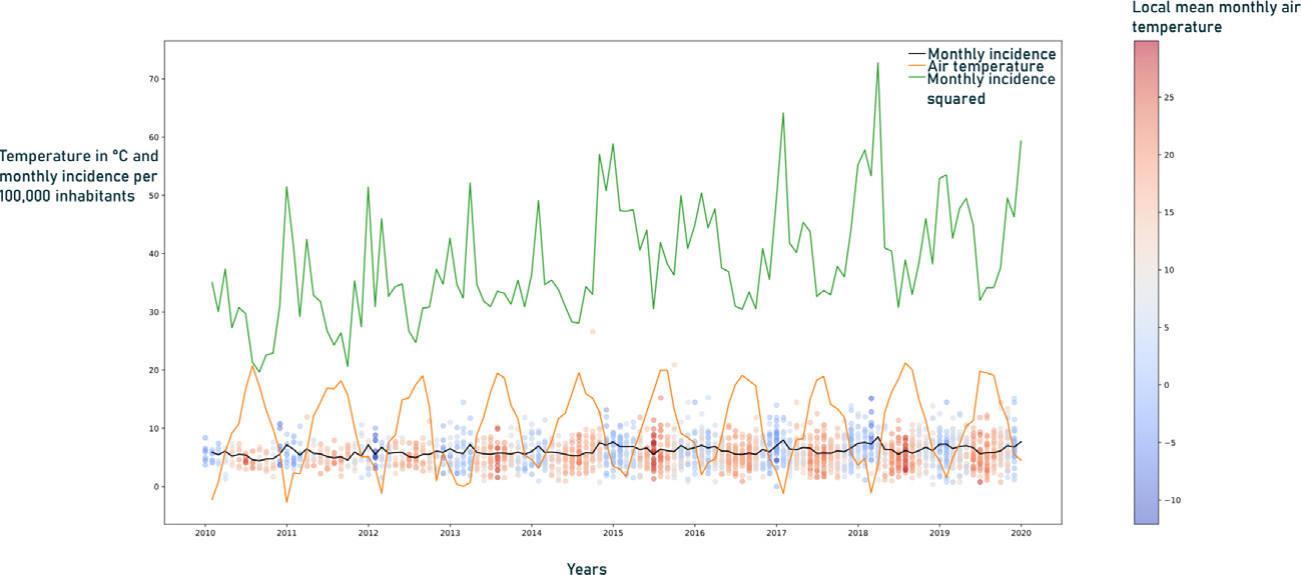
Incidence over time: The time series diagram illustrates the monthly incidence over time (black graph). The squared monthly incidence shows the effect more clearly (green graph). The average air temperature over the observation period is colored orange. The height corresponds to the temperature in degrees Celsius. Each round dot indicates an enclosed emergency medical service (station). The height represents the monthly incidence. The coloration corresponds to the average monthly air temperature. (For interpretation of the references to colour in this figure legend, the reader is referred to the web version of this article.)

The incidence of OHCA changes seasonal and periodic in Fig. 2. There is a significant and regular increase in the winter months. In months with moderate air temperatures and during the summer months, there is a drop in incidence. During summer months with elevated temperatures, such as 2015–2018 in Germany, we observe a slight rise in the incidence.

There was a significant increase in the monthly OHCA incidence on frost days from 6.39 (SD 95%: 6.36–6.41) up to 7.00 (SD 95%: 6.95–7.05, *p* < 0.001) per 100,000 inhabitants on frost days (Table 1). On summer days, the monthly OHCA incidence decreased significantly (6.61 [SD 95%: 6.58–6.63] to 5.79 [SD 95%: 5.73–5.85], *p* < 0.001 per 100,000 inhabitants).The results on high-humidity days showed a statistically significant increase in the monthly incidence of OHCA (6.43 [SD 95%: 6.40–6.45] to 6.89 [SD 95%: 6.83–6.95, *p* < 0.001]) per 100,000 inhabitants.

### Aetiology

On summer days, there is a trend toward more young people being affected (from 68.68 SD95%: 68.5–68.86 to 67.73 SD95%: 67.23–68.23 with *p* < 0.001). There are indications that more men could be impacted (from 64.8%/35.2% to 66.2%/33.8%), but not statistically significant (*p* = 0.062). On Frost days, there exists a trend wherein a greater number of elderly individuals are impacted (from 68.47 SD95%: 68.28–68.66 to 68.97 SD95%: 68.59–69.34, with *p* = 0.037). On days with high humidity, it there is no difference in age (*p* = 0.817).

### Outcomes frost/summer/humidity

On frost days, the number of deaths at the scene slightly increased (from 51% to 52.5%), while admission with ROSC decreased significantly (38.1–36.6%, *p* = 0.05). Survival to 30 days and patients with CPC 1 and 2 did not change significantly.

On summer days good and moderate CPC at hospital discharge did not significantly change (25–23.6%, *p* = 0.23) of all patients where a resuscitation attempt was performed, and further care was documented.

The results on high-humidity days the number of deaths on scene increased (51–52.7%), and admissions with ROSC decreased (38.1–36.3%, *p* = 0.022).

## Discussion

This study examined OHCA and the EMS in Germany, focusing on a population of up to 9 million inhabitants over ten years, comparing the number of cases involving resuscitation attempts with the local weather patterns. Meteorological days and an indicator (deviation from the usual local temperature of the month) were used as cohort dividers. Furthermore, we analysed the incidence, affected population group, and outcome of OHCA. Our study is the first to use data from a national resuscitation registry to investigate the impact of deviations from average ambient temperature on OHCA. The results suggest that in populations not adjusted to summer days and exposed to high humidity, the incidence of cardiac arrests may increase, thus supporting the trigger-temperature thesis. Cold temperatures seem to increase the incidence of OHCA and the chance of death at the scene and decrease favourable outcomes. The European Resuscitation Council’s 2021 guidelines recommend root cause research to improve the management of OHCAs. “Health systems should have population-based resuscitation registries that monitor the incidence, case mix, treatment, and outcomes of circulatory arrest. […] Data from registries should inform health system planning and responses to circulatory arrest.”^24^ Our study’s results contribute several findings in response to this call for research. Among other things, the GRR should be used to monitor data on environmental factor’s influence and impact on resuscitation, thereby preparing the ground for changes in resuscitation care planning and prevention like behavioural instructions for inhabitants.

Our findings show a significant increase in incidence on frost days and a progression in the number of cardiac causes associated with a rise in on-site mortality and a worsening of the chances of patient survival and overall outcome. We observed an increase in the incidence of OHCA in the group adjusted to the usual monthly temperature (G10: below the 10th and above the 90th percentile). Also, the outcome significantly decreased. To our knowledge, no other investigations are looking into the effects of people adjusted to local area weather phenomena. This is important for making better predictions and decisions for prevention. In high humidity conditions, there are significantly more deaths on the scene while the incidence substantially increases. According to the findings of Hensel et al., only humidity below 75% is positively correlated with the increase in the probability of OHCA.^6^ Tanigawa-Sugihara et al. also show a weak correlation with low humidity.^7^ Since we limited our investigation to days with a relative humidity of more than 90%, no statement can be made concerning days with low relative humidity. The inability to dissipate heat (high humidity) appears to lead to, among other things, hypovolaemia with reduced cardiac output, an increase in the haematocrit value, stress, and an increase in oxygen consumption.^13^, ^25^ Wehner et al. used the meteorological conditions of the fatal combination of a given threshold of heat and humidity that claimed several thousand lives in India and Pakistan in 2015 to illustrate their significant impact and the potentially deadly outcomes of such meteorological conditions.^16^ Therefore, we assume that high air humidity can have an unfavourable effect on the incidence and outcome of OHCA. The regression analysis (Supplementary Table 3) shows the dependency on temperature, as approximately every fourth case is linked to temperature (*R* = 0.273). However, it is interesting to note that only the cold days seem to have an effect, while the moderately warm days appear to decrease the monthly incidence. On summer days, we found a significant decrease in incidence. This observation could indicate that summer days as group separators that only reach a temperature above 25 °C once a day are an insufficient cut-off from moderate temperatures as a trigger range. This would align with the findings of Hensel et al., who showed that the probability of OHCA occurrence increased significantly at temperatures above 25 °C.^6^

Furthermore, this leads us to think that our dataset and timeframe do not contain sufficient hot days for further analysis. Moreover, it can be argued that days that impact OHCA have been rare in the past, at least in the timeframe and population studied. Based on the increase in the G10 cohort and as shown in Fig. 2, we assume that it may be possible that sweltering days could increase incidence.

### Strengths and limitations

When considering the implications of our findings, the limitations of retrospective studies must be considered. Due to missing outcome data, some data sets had to be excluded from further analysis. In addition, unknown factors and temporal shifts in the population may occur, for example, during the summer months due to holidays. Furthermore, the results may also be biased by unknown co-factors, while local weather phenomena could also play a role in this context. However, we attempted to minimise this by how the study was set up. In our study design, we have decided against analysing death on scene, as we aim to analyse all cases of OHCA where resuscitation was performed. Within this group, the time of death is unknown. This would add significant uncertainty to our study population. Our death on scene group cannot be used because it does not represent the real mortality in an area. There is no central register for data regarding the time and area mortality rate needed in Germany. In addition, while it was not possible to adjust for different approaches, guidelines, therapies, and local conditions, both prehospital and in-hospital, the examination of the monthly incidence yielded a large case base. However, in many locations, the monthly air temperature difference between summer and winter only amounts to 15 °C, flattening the results curve.

## Conclusion

In conclusion, we found a significant increase in the incidence of OHCA and worse outcomes for cold days. There was also a significant rise in the incidence of OHCA during days of high humidity. In addition, extreme temperature events for locally non-adapted regions also resulted in a statistically significant increase in incidence. Further research on this topic is needed.

## Funding

We have not received third-party funding.

## Data sharing

The data underlying this paper was made available under a data-sharing agreement with the GRR. Therefore, the data presented in this work can only be shared upon reasonable request and under the prerequisite that the GRR and its organising committee provide written consent.

## CRediT authorship contribution statement

**Maximilian Burger:** Writing – original draft, Visualization, Methodology, Investigation, Formal analysis, Data curation, Conceptualization. **Patrick Ristau:** Writing – review & editing, Investigation, Formal analysis, Conceptualization. **Andreas Bohn:** Writing – review & editing. **Matthias Fischer:** Writing – review & editing. **Ingvild Beathe Myrhaugen Tjelmeland:** Writing – review & editing. **Stephan Seewald:** Writing – review & editing. **Jan-Thorsten Gräsner:** Writing – review & editing, Supervision, Project administration, Funding acquisition. **Jan Wnent:** Writing – review & editing, Supervision, Project administration, Investigation, Conceptualization.

## Declaration of competing interest

The authors declare the following financial interests/personal relationships which may be considered as potential competing interests: ‘MB is a medical doctoral student supervised by JW and JTG. PR worked as a scientific coordinator for the GRR. AB, JTG, JW, MF, and SS are GRR’s steering committee members. IBMT works as a coordinator for the Norwegian Resuscitation Registry. AB, IBMT, JW and JTG are involved in the EuReCa studies. JTG is in the editorial board of Resuscitation.’.
