# Supplementary material for: Air temperature and humidity impact out-of-hospital-cardiac-arrests in Germany: A 10-year cohort study from the German Resuscitation Registry
Source: Resusc Plus. 2024 Aug 24;20:100750. doi: 10.1016/j.resplu.2024.100750 (PMC11387351; doi:10.1016/j.resplu.2024.100750)
Supplement: Supplementary Fig. 3 [file mmc1.pptx]

## Slide 1
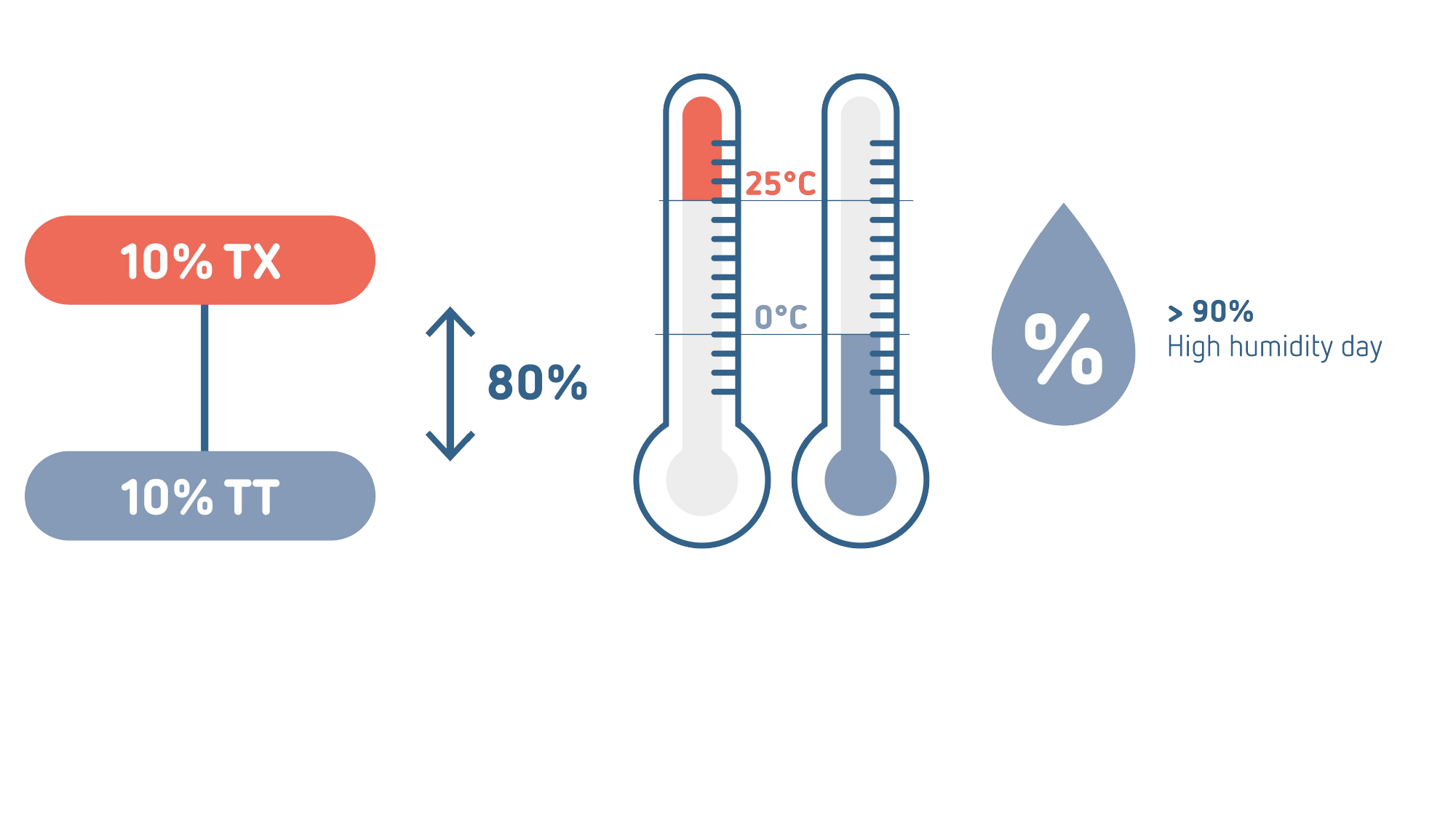

## Slide 2
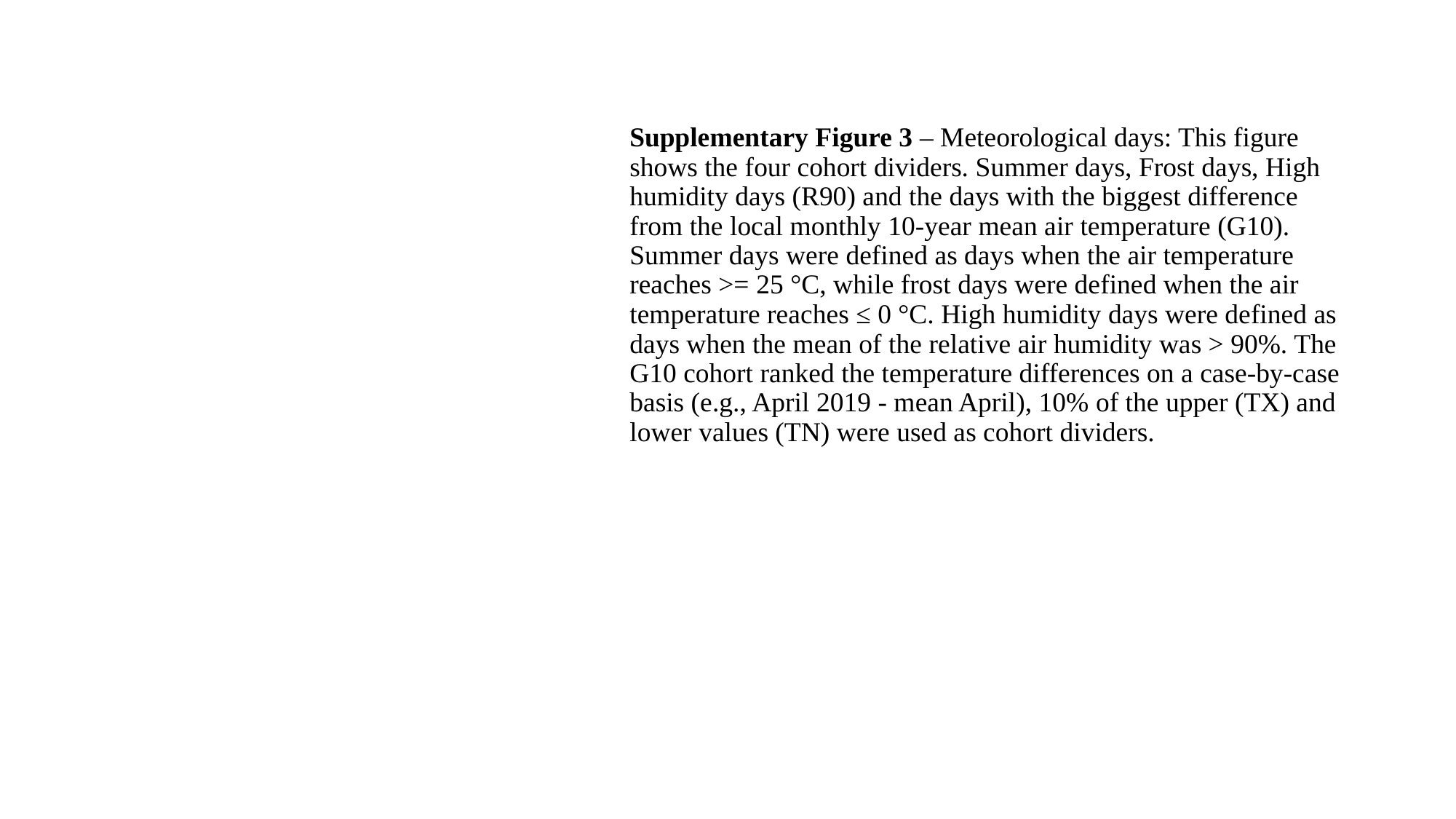

#
Supplementary Figure 3 – Meteorological days: This figure shows the four cohort dividers. Summer days, Frost days, High humidity days (R90) and the days with the biggest difference from the local monthly 10-year mean air temperature (G10). Summer days were defined as days when the air temperature reaches >= 25 °C, while frost days were defined when the air temperature reaches ≤ 0 °C. High humidity days were defined as days when the mean of the relative air humidity was > 90%. The G10 cohort ranked the temperature differences on a case-by-case basis (e.g., April 2019 - mean April), 10% of the upper (TX) and lower values (TN) were used as cohort dividers.
