# Supplementary material for: Air temperature and humidity impact out-of-hospital-cardiac-arrests in Germany: A 10-year cohort study from the German Resuscitation Registry
Source: Resusc Plus. 2024 Aug 24;20:100750. doi: 10.1016/j.resplu.2024.100750 (PMC11387351; doi:10.1016/j.resplu.2024.100750)
Supplement: Supplementary Tables 2 and 3 [file mmc2.docx]

**Supplementary Table 2** – Cases exposed to not exposed: Days with the biggest difference from the local monthly 10-year mean air temperature were named G10. The G10 cohort ranked the temperature differences on a case-by-case basis (e.g., April 2019 - mean April), 10% of the upper (TX) and lower values (TN) were used as cohort dividers.

| **Summer days** | | **Frost days** | | **High humidity days** | | **G10** | |
| --- | --- | --- | --- | --- | --- | --- | --- |
| Overall Cases: | 39094 |  | 39094 |  | 39094 |  | 39094 |
| Cases exposed: | | Cases exposed: | | Cases exposed: | | Cases exposed: | |
| non-extreme day | extreme-day | non-extreme day | extreme-day | non-extreme day | extreme-day | non-extreme day | extreme-day TN/TX |
| 34421 | 4673 | 31330 | 7764 | 32315 | 6779 | 31236 | 3943 |
|  |  |  |  |  |  |  | 3915 |

**Supplementary Table 3** – Regression analysis: SD95% stands for the 95% confidence interval.

|  | **Regression coefficient** | **SD95% lower-range** | **SD95% upper-range** | **Standardised coefficients** | **Significance** |
| --- | --- | --- | --- | --- | --- |
| **Constant** | 7·433 | 7·331 | 7·536 |  | ·000 |
| **Age** | ·002 | ·000 | ·003 | ·014 | ·006 |
| **Mean Monthly Temperature** | ·895 | ·808 | ·982 | 2755 | ·000 |
| **Monthly maximum temperature** | -·545 | -·590 | -·499 | -2008 | ·000 |
| **Monthly minimum temperature** | -·401 | -·444 | -·357 | -1029 | ·000 |
| **Difference from the local 10-year monthly average** | ·005 | ·004 | ·006 | ·046 | ·000 |
